# Help-Seeking Behaviors of Transition-Aged Youth for Mental Health Concerns: Qualitative Study

**DOI:** 10.2196/18514

**Published:** 2020-10-05

**Authors:** Chelsea Stunden, Julie Zasada, Nicole VanHeerwaarden, Elisa Hollenberg, Alexxa Abi-Jaoudé, Gloria Chaim, Kristin Cleverley, Joanna Henderson, Andrew Johnson, Andrea Levinson, Brian Lo, Janine Robb, Jenny Shi, Aristotle Voineskos, David Wiljer

**Affiliations:** 1 School of Population and Public Health Simon Fraser University Burnaby, BC Canada; 2 Office of Education Centre for Addiction and Mental Health Toronto, ON Canada; 3 Schulich School of Medicine & Dentistry Western University London, ON Canada; 4 Institute of Health Policy, Management & Evaluation University of Toronto Toronto, ON Canada; 5 Margaret and Wallace McCain Centre for Child, Youth & Family Mental Health Centre for Addiction and Mental Health Toronto, ON Canada; 6 Department of Psychiatry University of Toronto Toronto, ON Canada; 7 Lawrence S. Bloomberg Faculty of Nursing University of Toronto Toronto, ON Canada; 8 Health and Wellness University of Toronto Toronto, ON Canada; 9 Information Management Group Centre for Addiction and Mental Health Toronto, ON Canada; 10 Slaight Family Centre for Youth in Transition Centre for Addiction and Mental Health Toronto, ON Canada; 11 Education, Technology & Innovation University Health Network Toronto, ON Canada

**Keywords:** mental health, students, adolescent, substance abuse, eHealth, mHealth, mobile apps, help-seeking behavior, social stigma, social support

## Abstract

**Background:**

Transition-aged youth are particularly vulnerable to mental health problems, yet they are one of the least likely demographic groups to seek help.

**Objective:**

The aim of this study is to explore the influences on and patterns in help-seeking for mental health concerns among transition-aged youth who attend postsecondary schools in Canada.

**Methods:**

A qualitative research design was used, involving 12 semistructured focus groups with transition-aged youth (17-29 years) who attended postsecondary schools in Canada. A thematic analysis was conducted to code the transcripts and develop themes.

**Results:**

Four main themes and subthemes regarding the process and experience of help-seeking were generated: (1) the influence of formal service providers (accessibility and experiences), (2) the influence of social factors (system navigation and stigma), (3) the influence of health literacy (symptom recognition, acting on symptoms, digital tools and the internet, and mental health awareness campaigns), and (4) the influence of low-intensity sources of support, namely, self-help.

**Conclusions:**

Transition-aged youth seek help for mental health problems in different ways. Despite efforts to improve access to mental health services, transition-aged youth continue to face barriers to accessing these services, especially formal sources of support. The factors identified in this study that either hinder or facilitate help-seeking have pragmatic implications for developing help-seeking interventions and delivering mental health services for this population. In addition to other facilitators, family physicians are an important resource in the help-seeking process. Furthermore, digital help-seeking tools have unique characteristics that may make them an important source of support for transition-aged youth.

## Introduction

### Help-Seeking Behaviors of Transition-Aged Youth

The transition between adolescence and adulthood can be a particularly challenging time. Unique factors during this period, such as high levels of stress and anxiety, being introduced to alcohol and other drugs, and navigating new experiences and responsibilities, can make transition-aged youth vulnerable to mental health problems, which include substance use concerns [1]. Yet, they are one of the least likely demographic groups to seek help [2,3].

There is no universal definition of transition-aged youth. In the mental health literature, the age range spans anywhere between 14 and 29 years: some studies place it at 14-21 years, whereas others indicate 16-29 years [4]. For this paper, we adopted a broad definition of transition-aged youth of 17-29 years.

### Barriers to Help-Seeking

For this population, the process of seeking help for mental health concerns within the current social and health care system is arduous [5-7]. Barriers at the systemic, sociocultural, and personal levels can prevent or delay help-seeking among transition-aged youth [8,9]. A survey conducted in the United Kingdom found that 35% of youth with mental health concerns did not try to find help because of barriers such as poor access to care, perceived stigma, difficulty expressing concerns, and challenges navigating help-seeking processes or because they preferred to be self-reliant [10]. System-wide fragmentation makes it difficult to access appropriate services, information, and advice. These barriers have serious consequences, including self-medication and social, academic, and vocational difficulties [11]. They are compounded by other barriers such as long wait lists, stigma, high cost, lack of anonymity, and poor mental health literacy [12]. The result is often a failure to receive adequate mental health treatment [11-14]. The many barriers to help-seeking that transition-aged youth face need to be addressed to better serve the mental health needs of this population [8,9].

Eisenberg et al [15] identified 2 types of stigma associated with mental illness: perceived public stigma, which is defined as negative attitudes held by others, and self-stigma, which is defined as negative attitudes toward oneself. These 2 types of stigma interact and play a significant role in hindering help-seeking behaviors in transition-aged youth. Culture is another factor. Lack of cultural competence and sensitivity in mental health services, as well as the stigma attached to mental illness in many cultures, may make youths reluctant to seek help [15,16]. Together, these stigmas influence self-reliant behaviors, and transition-aged youths often prefer to cope with mental health problems on their own [1,2,16,17].

The nature of mental health problems can also be a barrier to seeking help. As these problems may have symptoms that are not easily recognized and often have a gradual onset, they might not be identified as issues that require medical attention; rather, the decision to consult a health care provider is influenced more by a voluntary help-seeking process [13]. Symptom recognition can be a factor in determining when to seek help.

### Facilitators to Help-Seeking

Although barriers to help-seeking for transition-aged youth have been well established in the literature, little is known about what facilitates help-seeking in this population [12,18]. Facilitators who have been identified include people having positive past experiences with formal and informal help-seeking as well as encouragement from others to connect with mental health services [12,19].

### Conceptual Help-Seeking Frameworks

In this paper, we use the conceptual framework by Rickwood and Thomas [13], which suggests that help-seeking is an adaptive coping process used to obtain external support to address a mental health concern. Within this framework, formal help is diverse and comes in the form of professional advice, support, and treatment delivered by professionals whose role it is to provide mental health care [13]. Semiformal help is offered by service providers who do not have a specified role in mental health care, such as teachers, coaches, and some community workers [13]. The third type of help, informal sources of support, such as friends and family, is also outlined in the framework and has been found to be important in encouraging youths to seek help [2,13,17,20,21]. Health Canada found that 27% of youth (aged 15-24 years) with mental, emotional, or substance use problems consulted informal sources compared with only 12% who consulted formal health professionals [20]. Self-help resources are a fourth type of support that includes sources such as self-help books and unguided website use. The framework also invites researchers to identify the stage within the process of help-seeking, the time frame, the type of help sought, and the type of mental health concern. Using a consistent framework to examine help-seeking is thought to increase the transferability of findings across studies and to better inform policy and practice decisions [13].

Confidentiality, trust, and positive relationships are important factors that encourage youths to pursue help from both formal and informal support groups [12]. Emotional competence (the ability to identify, describe, and manage emotions) can also predict help-seeking [17,22]. A survey of 300 undergraduate students found that those with low emotional competence were less willing to seek professional help and less likely to have positive experiences with mental health professionals [22]. More research is required to understand what factors promote help-seeking in transition-aged youth [8,9,23].

### Study Purpose

This study is part of a larger study that aims to evaluate and optimize a web-based and mobile health intervention called Thought Spot [24]. This crowdsourced digital platform enables transition-aged youth in postsecondary settings to look for and access mental health and wellness services. The app is a student-led project that prioritizes inclusion through steering committees, working groups, and focus groups. Thought Spot invites students to share their knowledge about services, discover wellness options on the web and in their geographical area, and post and read service reviews [24-26]. Although efforts have been made to improve access to mental health care for transition-aged youth, evidence suggests that barriers to care still exist [8]. The objective of this part of the larger study is to explore influences on and patterns in the help-seeking behaviors of transition-aged youth.

## Methods

### Setting and Participants

As part of the Thought Spot study [24], this paper reports the qualitative investigation of help-seeking behaviors that were generated through focus groups conducted throughout the study. A total of 17 focus groups were conducted on various topics as part of a participatory design research process to optimize the Thought Spot app. A subset of 12 focus groups that addressed help-seeking were examined for this paper. Participants were recruited using convenience sampling through web and offline methods that included posters, postsecondary school recruitment presentations, word of mouth, and social media posts (Facebook, Twitter, Instagram, and Slack). English-speaking youth aged 17 years or older who were enrolled in full- or part-time studies at a Canadian university or college were eligible to participate.

### Data Collection Procedures

Recruitment for this study took place over 3 years (July 2016-August 2018). Participants were enrolled on a first-come, first-served basis, with up to 10 participants per focus group. Priority was given to individuals who had not yet participated in the study. However, if there were fewer than 10 participants in a group, participants who had already attended another group were permitted to participate again if they requested to do so in response to study recruitment advertisements for subsequent groups. Participants were allowed to engage in more than one focus group, as each focus group was structured with different activities and modified help-seeking questions (Multimedia Appendix 1) [26]. In addition, we attempted to build engagement and capacity in the participatory design research process by having students participate and contribute more than once. Contributions of repeat participants were therefore expected to vary from group to group.

All participants signed informed consent forms and filled out a demographic form when they arrived for each focus group. A unique identifier was created based on participant demographics. Those who participated in multiple focus groups filled out a new demographic form for each group to avoid breaking confidentiality about having attended a previous group. Repeat participants were expected to use the same unique identifier each time. Participants received Can $40 (US $31) and bus tokens as remuneration.

A semistructured interview guide (Multimedia Appendix 1) was developed for the overall study, with questions focusing on seeking help and health information.

Interviews were conducted in English and audio-recorded. Nine master’s-level practicum students co-designed and facilitated the focus groups with the study team. There were 3 practicum student facilitators per group—1 acting as a note-taker, 1 as a flipchart assistant, and 1 as the main facilitator. These students included the authors (BL, CS, JS, JZ, and NV) and 4 students listed in the Acknowledgments section of this paper (AK, PD, SS, and VT). A staff research coordinator (AA or GF) and research analyst (EH) also attended the focus groups to provide general oversight and guide facilitation. Choosing to use practicum students to co-design and facilitate the groups reflected the decision to have transition-aged youth co-design the broader project. The focus group questions were broad and allowed participants to describe and discuss their experiences in as little or as much detail as they wanted.

### Data Analysis

Data collection continued until researchers determined that information saturation had been reached, as outlined in the published protocol [24]. Audio-recordings were transcribed verbatim, and data were entered into NVivo 12 (QSR International Pty Ltd) software after anonymizing and quality-checking transcripts for accuracy. Data were then coded using the 6 phases of thematic analysis by Braun and Clarke [27]. Phase 1 began with 1 researcher (JZ) reviewing the data and noting ideas as preliminary codes. In phase 2, 2 researchers (JZ and CS) generated initial codes based on all the transcripts and organized data relevant to each code. These codes included attributes of resources, types of service delivery, recommendations for future services, facilitators to use, barriers to use, outcomes of help-seeking, and help-seeking processes. In phase 3, 2 researchers (JZ and CS) organized data into potential themes based on the researchers’ understanding of the data and in relation to any categories that may apply from the conceptual framework by Rickwood and Thomas on help-seeking [13]. The themes were reviewed with a wider research team for input and feedback around grouping and conceptualizing the codes into themes. Updates to the themes included further refinements of the types of facilitators (eg, internal or external) and barriers to help-seeking (eg, concern with health care providers, costs, lack of social support, stigma, system navigation, time). The themes were then reviewed in phase 4 by 1 researcher (CS) to ensure that they worked in relation to the coded extracts, and the researcher also defined the final themes. The themes were reviewed and verified by the second researcher (JZ) in phase 5. Phase 6 involved finalizing the selection of quotes for analysis (CS) and relating the analysis back to the research question and then reviewing and confirming the analysis (JZ). The study received Research Ethics Board approval by the ethics boards at the Centre for Addiction and Mental Health, University of Toronto, Ryerson University, and George Brown College.

## Results

### Demographics

Participants were male and female aged 17 to 29 years who attended postsecondary schools in Canada. A total of 110 requests were received from students to attend the 12 focus groups examined for this paper (some students requested taking part in more than 1 focus group). Of these requests, 24 did not result in focus group attendance for various reasons. In 13 requests, participants signed up for a group but then canceled their subscription, 5 (39%) were ineligible, 3 (23%) were not available on the dates of the groups, 2 (15%) could not participate as the groups were full, and 1 (8%) did not respond after initial contact. The remaining requests resulted in 86 focus group attendees and 69 unique participants, based on our attendance data. A total of 10 participants attended more than 1 focus group, 2 attended 4 focus groups, 3 attended 3 focus groups, and 5 attended 2 focus groups. There was an average of 7 participants per focus group (range 4-9).

We were unable to remove 4 misidentified duplicate demographic surveys from our data. Therefore, although there were only 69 unique participants in the focus groups, we report the demographics of 73 respondents in Tables 1 and 2. The demographics of the focus group participants are listed in Table 1.

**Table 1:** The demographics of the focus group participants (N=73).

Demographics		Values, n (%)
**Gender**		
	Male	20 (27)
	Female	53 (73)
	Nonbinary	0 (0)
**Age (years)**		
	17-21	27 (37)
	22-24	32 (44)
	25-29	14 (19)
**Highest level of education achieved**		
	Some university but no university degree	27 (37)
	College diploma	6 (8)
	Bachelor’s degree	23 (31)
	Some graduate school but no graduate degree	6 (8)
	Master’s degree	4 (5)
	Other education or no response	7 (10)
**Ethnicity^a^**		
	Aboriginal (eg, First Nations, Métis, Inuit); Arab or Middle-Eastern; and Black (eg, origins include Canadian, American, Caribbean, African)	7 (9)
	East and Southeast Asian (eg, Vietnamese, Cambodian, Korean)	4 (5)
	Chinese	19 (24)
	South Asian (eg, East Indian, Sri Lankan)	18 (23)
	White or European	27 (34)
	Don’t know; other group	4 (5)
**Experience with mental health and substance use problems**		
	Yes	38 (52)
	No	26 (36)
	Don’t know or no response	9 (12)

^a^Some respondents answered in more than one category.

**Table 2:** Self-reported types of mental health or substance use problems (N=73).

Mental health problem^a^	Total, n (%)
Anxiety	19 (26)
Mood disorder (eg, depression, bipolar)	17 (23)
Comorbidity	16 (22)
Has a family member or friend with mental health or substance use problems	7 (10)
Borderline personality disorder, eating disorder, psychosis, posttraumatic stress disorder, and suicidal ideation	8 (11)

^a^Participants were able to indicate more than one answer.

All participants were identified as either male or female on the demographic form. *Trans* and *other* were also listed as gender options; however, no participants chose either of them. A subset (39/73, 53%) of participants elaborated on the types of mental health concerns they had (Table 2). To protect confidentiality and anonymity, if there were less than 4 responses for any of the categories in the demographics form, the responses were combined with other categories for the same question that also had less than 4 responses (Tables 1 and 2). This approach resulted in combined responses for some ethnicity categories and some types of mental health concerns.

Four main themes related to help-seeking were generated through transcript analysis and coding: formal services and providers influence help-seeking, social factors influence help-seeking, health literacy influences help-seeking, and self-help–low-intensity sources of support influence help-seeking. These themes and subthemes are listed in Multimedia Appendix 2.

### Formal Services and Providers Influence Help-Seeking

Formal sources of support are service providers with a specific professional role in delivering mental health (including addiction) care, such as family physicians, psychiatrists, and psychologists [12,13]. Participants discussed 2 main influences on seeking help from formal service providers: accessibility and empathy and trust.

#### Accessibility of Formal Service Providers

The ability of participants to access a formal service provider when they needed one was a common topic among the participants. Several participants reported contacting multiple formal service providers while help-seeking. They had difficulty accessing this type of support for various reasons. Participants often expressed concern about the instrumental costs (money and time) of seeking help from formal service providers. They also indicated that it was important during initial contact for service providers to create an empathetic and trusting environment. Establishing this connection made young people more willing to share their mental health concerns during subsequent encounters and seek treatment, services, or pathways to support in the future.

##### Wait Times Affect Access to Formal Services

Long wait times to be seen by formal service providers frustrated many participants and influenced their help-seeking behavior. One participant stated:

The wait times for getting any support are ridiculous no matter where you go. Oh, a year, two years, six months. I’m already in crisis right now and I want it right now or like a week from now, not a year from now.

Focus Group 1

Participants were also discouraged by the reality of having to consult multiple service providers to address specific aspects of their mental health concerns, a fragmentation that precludes holistic support and increases wait times. One student told us:

Everywhere you go…they want to only help you with one issue, it’s just not helpful.

Focus Group 1

When the desired treatment or support was not available in a timely way, participants sought alternative types of assistance, such as crisis services. One participant spoke about a friend:

If they were going through a crisis but they were waiting for the next appointment…they would always just go to the emergency department.

Focus Group 7

##### Financial Burdens Affect Access to Formal Services

Participants often described the financial burden associated with formal service providers (eg, psychologists, psychotherapists, counselors) in the community as a barrier to accessing help and pursuing optimal mental health. Some participants noted that although mental health services at postsecondary schools were generally more affordable than services in the community, these services sometimes make referrals to specialists in the community for students with serious mental health problems. Participants identified private psychotherapy as being inaccessible as it is expensive. They perceived pharmacological treatments to be more affordable. Accessibility was also limited by some health plans in Canada that did not cover participants’ preferred treatments. One student told us:

You often have to pay. And if you have no money or minimal money, there are not a lot of places that do scaling or they don’t inform you that they do scaling. So, if you want to see a social worker or a psychologist or psychiatrist, it’s often you need to pay $150 an hour.

Focus Group 3

##### Ease of Access Affects Access to Formal Services

The ease of access supported participants’ help-seeking efforts. Factors that increased access included convenient locations and times, anonymity and confidentiality, affordability, appointment booking, clearly displayed eligibility criteria, and short wait times. One participant stated:

I felt too anxious to really talk about things, so it really helped that they had an email address and I didn’t have to call in to make appointments, I could just schedule them over email.

Focus Group 7

Services and resources that provided a variety of options simplified the help-seeking process. Participants spoke highly of services that offered various entry points, multiple services, and several ways to learn about mental health. Some participants described their frustration with receiving siloed treatment for comorbid conditions and liked having services colocated in one place. One participant told us:

Agencies are becoming hubs now, so most places have everything on-site, so people don’t have to travel everywhere to get what they need.

Focus Group 5

Many participants identified family physicians as an important initial contact in the help-seeking process. Physicians helped them to navigate the mental health system and gave them information about other services or provided referrals to them. Family physicians typically made referrals to specialized mental health services. One student told us:

I think the easiest way is probably to go through a doctor or some sort of professional.

Focus Group 3

### Experiences With Formal Service Providers: Empathy and Trust

Participants discussed both positive and negative experiences with formal service providers that influenced their help-seeking behaviors. Negative experiences with health care providers and fear of similar experiences in the future deterred help-seeking. Many participants described a lack of empathy or understanding from health care professionals or had heard about others’ bad experiences, which made them hesitant to connect with these service providers. For several participants, the lack of connection made it difficult to talk about their problems. A student told us:

I don’t feel like I can trust another doctor. . .[They tell you] “It’s temporary. It will pass. It’s part of your life. You went through a crisis. It will go away. Don’t worry about it.” Or “I don’t believe you. Maybe come in a week and you’ll feel different.” That type of stuff. There’s a lot of, oh, oh. And if you do have mental illness, oh, maybe [you’re] lying. Maybe [you] actually don’t know what [you’re] talking about. So there’s that sometimes as well.

Focus Group 1

Negative past encounters decreased motivation to pursue help. Participants who had tried many times without success to access mental health support were skeptical about getting the help they needed or wanted. Some also lacked confidence in their current health care provider’s abilities or were dissatisfied with their care. Participants described feeling hopeless in these situations and being unable to seek help elsewhere because of long wait times or high costs. One student mentioned:

It’s hard to start over because if you lose the person you have, you’re going to have to start all over in the line. They’re not going to just give you someone else. You’re going to have to accept whatever you’ve got, and good luck trying to find someone else, because it took six months to a year to find whoever you got in the first place.

Focus Group 1

Participants also mentioned the importance of receiving feedback from peers before taking action. They wanted to hear about the experiences of others with a particular support before they invested their own time and resources. One participant said:

It kind of motivates you ... If someone else did this and found it helpful, I’m going to do the same thing as well.

Focus Group 3

### Social Factors Influence Help-Seeking

Social factors influenced help-seeking in both positive and negative ways. Participants discussed how informal supports (eg, friends, partners, parents) and semiformal supports (eg, teachers, work supervisors, academic supervisors, youth workers, coaches) could either facilitate or hinder help-seeking. Moreover, social stigma made it difficult for participants to speak about their mental health concerns and needs in their social networks.

#### Social Support Affects System Navigation

Social support networks were critical during help-seeking. Participants felt empowered when they received social support or encouragement or when they saw someone else succeed in getting help. One student mentioned:

I find a lot of times when someone describes an experience that they’ve had, I find that much more trustworthy than just looking at the idealized version of what it’s supposed to be.

Focus Group 3

Participants identified different chosen social support networks, and how useful they found these different support networks also varied. Some participants felt that they had limited support from friends or family. Some attributed this lack of support to attending school far from home. Others mentioned that social isolation was connected to experiencing more severe symptoms of mental illness. Both situations hindered help-seeking. Complying with treatment was particularly difficult for participants who did not have family support. One participant told us:

Sometimes…you ask for help and your family says we’re not going to help you with that…You’re prescribed medication and…your parents won’t give you the money you need to buy them. But then your counselors are also like, “Well, you can’t not take the medication.”

Focus Group 7

Other participants had friends or family who were able to recognize and respond constructively to their mental health problems and facilitated the help-seeking process on their behalf. One participant said:

So, normally in high school…at least in my experience, if you’re struggling with mental health issues, your parents will kind of handle it. Like, your parents will make your doctor’s appointments and find referrals and find you a psychologist or a psychiatrist. And by [post-secondary school], you have cycled through the system enough times that you’re fairly familiar and you know what to seek out. However, I know a lot of people who started university and, in their first year, developed some mental health issues. A lot of friends of mine would ask me, because they knew that I had experience with that, and they just feel lost.

Focus Group 8

For some, informal social supports were the first step to finding help. These people helped participants navigate the process and shared their own experiences. A student told us:

We went online, but we found the online help to not be very specific or applicable and [we] had to take it one step further and consult either a friend or somebody who has been through it to get more of the personal touch.

Focus Group 7

Semiformal supports who do not have a specified role in mental health care (eg, coaches, residence fellows, professors) also helped participants navigate the system and checked in with them to see whether they had followed through on their intended action. One student told us:

I’ve seen profs facilitate with counselors, making sure that the student is able to find someone or know where to go. . .If you tell someone and they’re able to help you through the process and just support you through it, then makes it a lot easier.

Focus Group 1

#### Stigma Affects Help-Seeking

Stigma was reported as a deterrent to seeking help from formal and informal supports. Not all participants had social networks that offered support around seeking help for mental health concerns. One participant mentioned:

Within my group of friends. . .we don’t talk about mental health that much, if at all.

Focus Group 3

Participants discussed the stigma of mental health problems and their fear of discrimination if they disclosed their mental health concerns. Internalized stigma, feelings of shame and embarrassment, and being in denial about their situation were common experiences. One participant said:

I think sometimes seeking help can also be like a sign of weakness and that kind of stops you from going to seek help. Like, you feel like you should be able to deal with these things by yourself.

Focus Group 9

Participants were often afraid of being judged by friends, peers, and family. One student told us:

Being involved in a group of friends that actually make fun of mental health problems, you can’t even talk about it.

Focus Group 7

Another student said:

Before, I had to go through my parent’s insurance plans and they would know what I was taking all the time and it would make me feel uncomfortable and judged.

Focus Group 7

Participants also feared discrimination by their professors and peers and the potential consequences of disclosure for their career or academic ambitions. One participant said:

I’m not sure if telling my personal or health issue to my prof will actually. . .what if I need a recommendation letter from this person later? Will this color their opinion of me and my work abilities?

Focus Group 1

One participant described how the stigma attached to pharmacological treatment made a friend hesitant to seek help:

It’s not just the side effects that he was worried about. He was also worried about the stigma associated with having to take medications for his depression.

Focus Group 1

Participants who were trained to be mental health service providers (eg, social workers, medical students, residents) also spoke about the unique stigma associated with help-seeking as a mental health care provider. One student in social work told us:

I’m in social work. You’re supposed to be able to help people. You’re not supposed to be the sick one too...You’re not supposed to be your clients…even though maybe those that need the help are those that are providing the help as well. And it’s sometimes fearful for those that are providers just because they are told that they’re not supposed to [seek help from formal health service providers] . . . because then they won’t want to hire you.

Focus Group 3

As a result of these concerns, many participants wanted to keep their mental health problems private and seek help on their own. One participant said:

I think it’s still taboo to talk about getting help for mental health or addictions. There’s more access now, but I think. . .people don’t like asking for help.

Focus Group 1

Although societal and internalized stigma made some participants reluctant to seek support from their social networks, several participants spoke about the importance of finding people they could relate to. One student told us about a friend’s experience:

After she was diagnosed and everything, she told one friend about it and from there it just started a chain reaction, and one by one, everybody started saying. . .“Oh, we have that problem too, but we were too scared to tell anybody about it because it’s such taboo with our parents.”

Focus Group 1

### Health Literacy Influences Help-Seeking

Participants discussed factors related to health literacy that could influence help-seeking. Mental health literacy is an awareness and maintenance of one’s own mental health needs, becoming knowledgeable about formal diagnoses, treatments, and the effect of stigma, and being able to effectively seek help when needed [28]. In this study, steps toward health literacy by participants included recognizing symptoms, acting on symptoms, learning about mental health through web-based sources or awareness campaigns, and finding out what services are available. Participants used multiple entry points to learn about mental health problems and the types of services they could access.

#### Health Literacy Affects Symptom Recognition

Participants described difficulty in identifying mental health concerns and attaining and maintaining positive mental health. They noted that they needed first to be self-aware and recognize that their psychological state was out of the ordinary before they would access services. One participant said:

I think a big thing was. . .self-awareness and this realization that maybe. . .this is not how you should be feeling every single day. That’s like a very big turning point where you realize that. . .it’s not normal to feel this way every day and you shouldn’t have to feel this way every day.

Focus Group 3

Lacking this insight or not understanding how to find help, which includes getting a diagnosis, made it difficult to search for appropriate services. One participant told us:

The very first step is to get a diagnosis. However, it’s very difficult for people to take that step. Like, I genuinely do not know what services or programs are available for people who are just kind of experiencing symptoms but don’t have a diagnosis. . .because. . .a lot of places. . .only provide these programs and services for people who already have a proper diagnosis.

Focus Group 8

Several participants also explained that youths might not know what types of assistance are available and need help to find appropriate resources or support. One participant said:

Yes, it’s hard to define what mental health help actually is. And so people tend to [run] in circles, avoid conversation around it because they don’t really know how to describe what it is.

Focus Group 2

Some participants described not always being able to articulate their mental health history and symptoms, which made it difficult to solicit support from their networks. One participant said:

I feel like maybe sometimes you’re sitting at the doctor’s office and you’re probably experiencing some. . .mental health problems that you also want to talk about, [but] you don’t know how to talk about it, and then time’s up and the doctor needs to move on to the next patient, and then it kind of falls through the cracks.

Focus Group 1

#### Symptom Recognition Does Not Always Lead to Help-Seeking

Although recognizing symptoms was the first step in seeking help, self-awareness did not always lead to action. Some participants described being aware of their distress but downplaying it or not knowing when to seek help. One participant told us:

I find I always do this thing where I end up looking at someone that’s worse off and I’m like, I can probably handle it or I can probably deal with it.

Focus Group 9

Another participant said:

Participants also described lacking the motivation to pursue help, because of the symptoms of their mental health problem and “feeling so hopeless that you don’t even want to seek [help].”

Focus Group 2

Participants who recognized that something was wrong described weighing the pros and cons of getting help. Some participants eventually accessed services as certain stressors made them feel that their situation had evolved into something more serious and they could no longer avoid getting help. One participant said:

I’ll never be feeling fine and thinking, “Oh, I should probably worry about my mental health right now.” It’s usually when I’m already at that low point, where I’m just desperately seeking mental health services.

Focus Group 7

#### Health Literacy Is Affected by Digital Health Tools and the Internet

Accessing digital health resources on the web increased participants’ mental health literacy. Participants often sought information through web-based media to validate their feelings and identify what kind of help was available for their particular concerns. One student told us:

I’d go on [website]. . .it’s not a diagnosis, but it can help you seek out one. I found that what I got was essentially what the doctor told me.

Focus Group 8

Using mental health resources effectively required being able to match information or services to identified needs and then feeling comfortable and confident reaching out to these supports. Participants often described web-based resources as an important entry point to help-seeking. One student said:

I feel like the internet can be a good stepping stone. It’s not really somewhere that you find conclusive answers, but it’s a good starting point.

Focus Group 7

Participants often described using the internet and social networking websites or mobile apps (Facebook, Instagram, and YouTube) to look for support and mental health information. One participant said:

There’s a lot of Facebook group communities that sometimes are available. Not really services, but, like, groups of people that are like, “Yeah, I have the same problems.”

Focus Group 8

Participants spoke about learning from the experiences of others. One participant said:

I’ve also seen different videos, where a lot of YouTubers that I used to watch or I sort of watch, they’ve also gone through mental health issues. So a lot of them post about their experiences…Seeing someone open up about an experience and maybe you can relate, then it really takes off a lot of stress.

Focus Group 9

However, web-based information about mental health could be confusing, so participants sometimes reached their own conclusions. One student told us:

A lot of different mental illnesses have the same symptoms…You end up self-diagnosing yourself with a bunch of different illnesses, which you might not have all or any of them.

Focus Group 9

Participants who sought support or resources on the web also described the information as ambiguous and the amount of information as overwhelming. One student said:

It definitely looks like there’s a lot of resources out there for a variety of needs and lots of stress[ors] people might be feeling, but. . .first being able to find and decide upon which one you actually want to pursue takes a lot of time. Then, when you actually try to access it, it seems really difficult and daunting.

Focus Group 9

Despite the issues that participants highlighted about using web-based resources, digital media was a popular source of help-seeking.

#### Health Literacy Is Affected by Mental Health Campaigns

Participants reported that mental health campaigns improved their mental health literacy. Social marketing and other mental health campaigns increased motivation to seek help. Participants described feeling empowered by hearing other people’s stories about finding support. One student told us:

Bell Let’s Talk Day comes around, and [people] start posting all over Facebook. . .being like, “I started out on this and got more comfortable with it, and now I’m better and I want to help other people get better.”

Focus Group 7

Other participants recalled learning about support through promotional events, guest speakers, emails, posters, and pamphlets in high school. One student said:

We had a social worker in high school that would come to all of the classrooms, so you were getting to see a social worker regardless.

Focus Group 4

Some participants recalled learning about mental health services before they personally experienced mental health symptoms. They became aware of these services through information sessions, word of mouth, emails, posters in university common rooms, pamphlets at community centers or health offices, advertisements on websites, or in-course syllabi offering advice or support for mental health and wellness. Many participants mentioned a phone helpline that was advertised on cereal boxes. Knowing about mental health resources before they were even needed supported navigation by providing a good starting point. One student told us:

Even before I had to use the service, I was aware of what it was. Even before I had an inkling that I would ever need to call, I just knew what it was.

Focus Group 4

### Self-Help: Low-Intensity Sources of Support Influence Help-Seeking

Participants reported trying to take care of their mental health concerns on their own and discussed the importance of resources that enable self-management. They described self-help and wellness activities for managing mental health concerns as generally low-cost, self-guided, and immediately available. Participants who were not comfortable accessing crisis or formal services found self-help particularly useful.

Participants often experimented with making small changes in their lives through self-help apps that do not involve live support (eg, e-learning, web searching, computer-mediated therapy, mindfulness sessions). One student said:

On YouTube. . .it’s not actually going to see someone for cognitive behavioral therapy, but there’s resources online of learning some of the techniques that they use and maybe they can implement, even if they don’t actually go seek help [formally].

Focus Group 4

Another student mentioned:

They had these online Instagram things where every day of the week it was a day of self-care.

Focus Group 2

Participants also accessed various digital mental health resources, such as Headspace [29], Meditation Studio App [30], 7 Cups [31], Buddify [32], and Virtual Hope Box [33]. Participants used these apps to manage their mental health every day, track patterns in their mood, or chat anonymously with people about their mental health concerns. One participant said:

People who are in situations that don’t have a lot of time – students, mature students, parents, etcetera – use online stuff like 7 Cups.

Focus Group 5

Unlike participants with more urgent mental health care needs, those who had only mild or moderate symptoms felt that crisis-based services were not a good fit for their needs, although they seemed to be the most readily available and accessible. One participant said:

When I think of a hotline, I think of like an emergency or dire situation. It’s not something that comes as a first resource for me.

Focus Group 9

Instead, participants with less urgent concerns tried to meet their needs through other accessible and affordable support for overall health and wellness (eg, fitness, meditation and mindfulness, journaling, nature walks, playing music, listening to music, playing with pets). One participant told us:

[I do] hobbies that require a lot of concentration. If you play a musical instrument, you’re forced to focus on the instrument and the music, and then your mind just completely forgets what it was stressed about at the time, so it kind of takes your mind off that, and when you come back to that situation, you kind of see things in a new perspective.

Focus Group 3

Other wellness resources were also common. Some participants temporarily distracted themselves from stress and unpleasant tasks with *guilty pleasures*, such as watching YouTube or Netflix, or playing video games to clear their minds. However, they recognized that these activities provided only a temporary reprieve. One participant explained that Netflix sometimes reduced productivity as it was easy to spend a lot of time on it and neglect important tasks. One student said:

Netflix is a common thing now. I think everyone watches a lot of TV. . .But, if you’re obsessive, like watching massive amounts of TV, then it can really make things worse by procrastination of everything.

Focus Group 3

Although participants identified many low-intensity sources of support, they recognized that some activities that youths use to cope with difficult feelings or situations may not be healthy. One participant said:

Professionals, they’re not really going to recommend going out for drinks, but sometimes going out for drinks just helps you deal with it.

Focus Group 8

Other participants pursued wellness by accessing low- or no-cost community- or school-based mental health resources. Alternatives to crisis services or professional support are listed in Multimedia Appendix 3.

## Discussion

### Principal Findings

Despite the high prevalence of mental health problems that develop in adolescence and early adulthood, many young people still do not seek help [10,34-36]. This study identified many of the barriers to and facilitators of help-seeking that previous research has found, such as accessibility of sources of support, experiences, system navigation, stigma, symptom recognition, and behavioral intention. It also offers new insights for developing strategies and services to meet the mental health needs of transition-aged youth by discussing the role of digital resources in the help-seeking pathway and the types of self-help resources that young people look for during this process. We found that help-seeking differs significantly among transition-aged youth in terms of the types and forms of support they prefer. Many use a combination of support, which can be categorized into formal, informal, and self-help support, using the conceptual framework by Rickwood and Thomas [13]. Our study contributes to the existing literature by identifying a range of self-help options beyond websites that were accessed by youth. We added a new category for digital self-help apps that offer peer support, therapeutic techniques, or other forms of self-help (Multimedia Appendix 3) because they represent a fairly recent distinctive self-help category not included in previous classifications. Identifying these types of support and categorizing them according to a conceptual framework is an area for ongoing development within the help-seeking literature, in which most types of assistance are not adequately described or classified [13].

### Frequent Use of Low-Intensity Services During Help-Seeking

Our study suggests the frequent use of low-intensity services, such as the web or mobile apps, among transition-aged youth. These digital services were noted as particularly helpful because of their ease of access. Our findings support other research that identifies web-based mental health resources as a primary source of support for transition-aged youth [13]. Many participants in our study reported using educational websites about therapeutic techniques, peer-to-peer active listening services, interactive self-help mobile apps, and social media (eg, Facebook, Instagram, Yik Yak). Recreational activities, accessed through websites such as Groupon and Meetup were also mentioned as ways to support mental health and overall wellness. The range of digital self-help apps that the youth in our study accessed are listed in Multimedia Appendix 3. Our findings add to the current literature by suggesting that popular digital mental health and wellness apps may be unique access points for reaching young help-seekers. Improving and expanding these resources should become a priority, particularly where traditional offline mental health services are difficult to access [37]. More research is needed to determine the effectiveness of these emerging types of support.

### Web-Based Health Information May Hinder Help-Seeking

Self-directed efforts to increase health literacy also influenced the help-seeking process. Digital health solutions and the internet can facilitate access to information about mental health problems and services and increase mental health literacy. However, participants in our study cautioned that web-based self-help resources can also be counterproductive if users are unable to decide which resources match their needs. We reiterate other researchers’ cautions that the web can cause information overload and increase the potential for harm (such as incorrect self-diagnoses) when help-seekers lack the skills and experience to evaluate the information they find on the web [12,38,39]. New coordinated public health initiatives should focus on teaching transition-aged youth how to evaluate the quality and credibility of web-based health information.

Although seeking help on the web was common, it was not always helpful, and the credibility of sources was sometimes uncertain. Youth also accessed emotional support on the web where they could anonymously engage with a community of peers who offered support by sharing points of view and helped them decide what help to pursue. Web-based help-seekers read testimonials and web-based reviews about what to expect from each service provider.

### Social Marketing Campaigns and Educational Outreach May Catalyze the Help-Seeking Process

In addition to actively pursuing help, transition-aged youth are also learning about mental health supports through social marketing campaigns and educational outreach. Research has found that youth who are exposed to social marketing or educational outreach around mental health are more likely to reach out for help and get treatment [40,41]. Our research adds to these findings, suggesting that early educational intervention has long-term positive effects on help-seeking in young people. More research is needed to determine the most effective way to encourage transition-aged youth to seek help for mental health problems.

### Instrumental Barriers to Help-Seeking

The findings of our study are also consistent with research that shows that general mental health care is largely inaccessible because of instrumental barriers that include distance, cost, and wait lists [42,43]. Although on-campus services at postsecondary schools in Canada can mitigate some of these barriers, for example, they are usually supported by tuition and ancillary fees or are covered by student insurance, these services also often have long wait times. Additionally, walk-in appointments and drop-in sessions are not always offered. Despite the advantages of campus-based services, our findings suggest that barriers remain for transition-aged youth who seek services on and off campus.

The extensive wait times reported in the literature were echoed in our findings. Only about 8.6% of agencies in Canada do not have a wait list for child and adolescent programs or services. However, timely access to treatment is critical because long wait times not only prolong psychological and physiological discomfort but can also increase attrition rates, reduce motivation to seek help, and decrease expectations and positive treatment outcomes [44,45]. Barriers to accessing care are compounded by other barriers, such as service fragmentation, poor health literacy, low motivation to change, and lack of social supports. Together, these barriers lead to underused or inappropriate services, poor follow-up rates, decreased service quality, and high health care costs.

### Mental Health Service Providers Influence Help-Seeking

Previous experiences with formal service providers were either deterrents or facilitators to help-seeking, particularly given the challenges of navigating the fragmented mental health care system. Past help-seeking experiences that were empathetic and instilled trust encouraged future help-seeking. Similarly, negative, stigmatizing experiences discouraged help-seeking. Our findings contribute to existing evidence that positive experiences with formal health providers, which focus on building a trusting relationship, are important to youth [12].

Family physicians are increasingly being recognized for their role in the mental health help-seeking process by supporting transitions between youth and adulthood [46], providing treatment [47], and making referrals to specialists [17]. Transition-aged youth in our study primarily viewed family physicians as gatekeepers for referrals to mental health specialists. Other studies have also found family physicians to be the first point of contact and to be responsible for making referrals, despite facing numerous barriers themselves (eg, lack of time, knowledge, resources, training) [47-50]. Our findings reiterate the unique role of family physicians in help-seeking and point to the need to train and support family physicians in guiding young patients through system navigation and treatment. Significant benefits could result from developing supports for family physicians to manage and diagnose young people with mental health concerns by offering timely access and continuity of care.

### Help-Seeking for Mental Health Providers in Training

Transition-aged youth in our study who were undergoing training to become formal mental health service providers, such as social workers or physicians, described a unique barrier to help-seeking in the form of stigma. As future professionals whose responsibility is to help others, they feared that disclosing their own mental health problems would damage their reputation and career prospects. However, mental health problems among health care professionals are well-documented and include depression, anxiety, substance abuse, and suicide risk [51-55]. Other research suggests that emotional distress during training (eg, medical residency) can persist into professional practice and lead to burnout [56]. To our knowledge, no research has been conducted on the help-seeking behaviors of this population; however, our findings underscore the importance of offering formal mental health supports tailored to the needs of mental health care providers in training and point to the need for further research in this area.

### Limitations

The qualitative approach of this study enriches our understanding and insights into the mental health help-seeking behaviors of transition-aged youth; however, the findings may not be generalizable beyond the Greater Toronto Area, where the study took place. Help-seeking behaviors likely differ across postsecondary and cultural contexts, particularly at postsecondary schools that do not have access to on-campus support services. In this study, it was not possible to determine the effects of location, sex, gender, or culture on help-seeking behavior. Our findings are useful for health care providers who are creating digital mental health programs and services to improve access to care. Using focus groups provided an opportunity for participants to express their personal experiences while navigating the help-seeking process. However, a limitation of focus groups is the potential toward normative discourse, especially in the context of mental health where people may not be comfortable talking about certain issues in a group setting. Another limitation was that certain groups were underrepresented in this study: depression and anxiety were the mental health problems reported by the vast majority of participants, and most participants were female. Youths with serious mental health problems (eg, psychotic disorders) and transition-aged males are known to be less likely to access any sort of health care or to participate in research [57]. These groups may be better engaged in their help-seeking behaviors through other channels, such as technology, health clinics, schools, and social media [58].

Multiple focus group attendance by participants who requested it was another possible limitation in this study (10 participants attended more than 1 group) as it has the potential to amplify some themes in relation to others. The rationale for agreeing to these requests was to increase participants’ overall, sustained engagement with the participatory design research process throughout the study. As described in the Methods section, because of the differences between focus group topics, the contribution of participants was expected to be different from group to group. Another limitation related to repeat participants, who filled out a demographics form each time they attended a focus group, was that we were unable to remove 4 misidentified duplicate demographic surveys from our data. This resulted in reporting on demographics for 73 participants, although only 69 unique participants were involved.

### Conclusions

Transition-aged youth are influenced by various barriers and facilitators when they seek information and support for mental health problems. Future investments should focus on reducing barriers associated with accessibility, system navigation, stigma, and symptom recognition. This study also identified digital health tools, the internet, and low-intensity sources of support as important parameters in the help-seeking process. Family physicians were found to be important gatekeepers to other mental health services. Digital and web-based technologies and the additional supports or features they offer may also be powerful tools for promoting help-seeking. Young people who are trained to become mental health care providers may experience a unique barrier to help-seeking in the form of stigma, an area that warrants future research. There is also a need for research on the effectiveness and unique features of low-intensity supports in seeking help for mental health problems.

## Appendix

Multimedia Appendix 1 Semistructured interview guide.

Multimedia Appendix 2 Themes from qualitative analysis.

Multimedia Appendix 3 Mental health supports discussed by study participants.
