# Supplementary material for: Help-Seeking Behaviors of Transition-Aged Youth for Mental Health Concerns: Qualitative Study
Source: J Med Internet Res. 2020 Oct 5;22(10):e18514. doi: 10.2196/18514 (PMC7573698; doi:10.2196/18514)
Supplement: Multimedia Appendix 1 [file jmir_v22i10e18514_app1.docx]

Supplementary File 1

## Semi-Structured Interview Guide

### Help-Seeking

How is accessing mental health services, or asking for mental health or substance use support, perceived by your friends?

1. Is it openly discussed? Why or why not?
2. Is there push back? Is there stigma involved? Why or why not?
3. What types of support or services have your friends needed? Did they get the support or services that they needed?

If you were experiencing emotional or psychological distress who would you talk to or where would you seek help from?

1. What are some factors that would make it harder, or would stop you, from seeking help?

What are some factors that would make it easier for you to seek help?

Have you ever searched for mental health, substance use or wellness services for yourself or for someone else? What was your experience looking for this information?

1. Where did you look for this information? (eg, on campus, online, books, pamphlets, family, friends, professionals)
2. Where did you FIRST look for this information?
3. Did you find the services or support you were looking for?
4. If yes, what helped you find these services?

If no, what were the barriers to you finding the services or support you were seeking? What made finding the information difficult?

How do you normally look for health and/or wellness services?

Do you use the same approach to searching for health care services (eg, mental health counseling, substance use support, sexual health clinics) as wellness services (eg, yoga classes, outdoor spaces, academic support)?

### Health Information–Seeking

Have you ever searched for information about mental health or addictions for yourself or for someone else? What was your experience looking for this information?

1. Did you find the information you were looking for?
2. If yes, what helped you find the information?

If no, what were the barriers to you finding the information you were seeking? What made finding the information difficult?

What are the main sources of mental health and substance use information you rely on? (ie., online, books, pamphlets, family, friends, professionals?)

1. If you wanted to find mental health and substance use information, what would your process be for finding this information you need?
2. What types of mental health and/or substance use information do you look for when seeking information?

How would you use the information you have found?

In general, if you were seeking health information online, which websites/resources/mobile apps would you access first? Why?

1. How would you look for these?
2. How would you determine whether the app, website or information is helpful?
3. How would you determine if the health information is accurate or reliable?
4. How do would use the health information you find?
