# Supplementary material for: Help-Seeking Behaviors of Transition-Aged Youth for Mental Health Concerns: Qualitative Study
Source: J Med Internet Res. 2020 Oct 5;22(10):e18514. doi: 10.2196/18514 (PMC7573698; doi:10.2196/18514)
Supplement: Multimedia Appendix 2 [file jmir_v22i10e18514_app2.docx]

# Supplementary File 2

## Themes from Qualitative Analysis

| Former Services and Providers Influence Help-Seeking   - Accessibility of Formal Service Providers - Wait times affect access to formal services - Financial burdens affect access to formal services - Ease of access affects access to formal services - Experiences with Formal Service Providers: Empathy and Trust |
| --- |
| Social Factors Influence Help-Seeking   - Social Support Affects System Navigation - Stigma Affects Help-Seeking |
| Health Literacy Influences Help-Seeking   - Health Literacy Affects Symptom Recognition - Symptom Recognition Does Not Always Lead to Help-Seeking - Health Literacy is Affected by Digital Health Tools and the Internet - Health Literacy is Affected by Mental Health Campaigns |
| Self-Help: Low Intensity Sources of Support Influence Help-Seeking |
