# Supplementary material for: Help-Seeking Behaviors of Transition-Aged Youth for Mental Health Concerns: Qualitative Study
Source: J Med Internet Res. 2020 Oct 5;22(10):e18514. doi: 10.2196/18514 (PMC7573698; doi:10.2196/18514)
Supplement: Multimedia Appendix 3 [file jmir_v22i10e18514_app3.docx]

# Supplementary File 3

## Mental health supports discussed by study participants

The following resources are categorized into formal, informal, semi-formal, and self-help sources of support, according to Rickwood & Thomas’s [13] conceptual framework for help-seeking. We have added a category for digital self-help apps because they seem to represent a fairly recent distinctive self-help category not included in previous classifications. We have classified resources according to our local context, following recommendations that classifications could differ according to contexts and other factors [13].

Rickwood & Thomas’s [13] classification of sources of support follows:

Formal: Professional health service providers with a specified role in delivery of mental health care, ie, psychiatrist, psychologist, general practitioner, mental health nurse

Semi-formal: Service providers and professionals who do not have a specified role in delivery of mental health care, ie, teacher, work supervisor, academic advisor, youth worker, coach

Informal: ie, friend, partner, parent

Self-help resources: ie, unguided website use

| Formal | Informal | Semi-Formal |
| --- | --- | --- |
| Counselling services centre  Crisis line  Drop-in clinic  Family physician  Good2Talk  Group therapy  Health and wellness clinic  Hospital (CAMH)  Kids Help Phone  Professional health association  Social worker | Friends and family  Professor | 211 (Community and Social Services Help Line)  416 Community Support for Women  Community centres  eCounselling  Good Shepherd  Peer-based services  Youth programming  Workshops  Toronto Distress Centre |
| Self-Help | Digital Self-Help Apps |  |
| Art exhibition  Eventbrite  Youtube  Facebook  Fitness centre  Google  Groupon (e.g.: for discounted services)  Instagram  Journaling  Listening to music  Massage  Meditation class  MeetUp.com  Mindfulness session  MySpace  Parks; dog parks  Pet therapy / playing with pets  Place of worship  Playing music  Netflix  Reddit  Buzzfeed  Listpay  Social events  Video games  Walks in nature  WebMD  Wikipedia | 7 Cups  Bik  Blog therapy  Buddify  Carrot Rewards  -The Happiness Planner  HeadSpace  HopeBox  Meditation Studio  -Yik Yak  Computer-mediated therapy |  |
